# Computational study on B, N, and Si-doped C60 nanocages for acetone detection in heart failure diagnosis and environmental remediation

**DOI:** 10.1038/s41598-025-27880-3

**Published:** 2025-12-16

**Authors:** Abdulwahab Alamri, Ahmed Alafnan

**Affiliations:** Department of Pharmacology and Toxicology, College of Pharmacy, University of Ha’il, 55476 Hail, Saudi Arabia

**Keywords:** Quantum Chemistry, Medical Physics, Acetone detection, Environmental pollutant, Heart failure, Sensor, Chemistry, Environmental sciences, Materials science, Nanoscience and technology

## Abstract

Acetone is a volatile organic compound that acts both as an environmental pollutant and as a biomarker for metabolic disorders such as heart failure. Therefore, early and sensitive detection of acetone is of great importance for environmental monitoring and medical diagnosis. Recent advances in carbon-based nanomaterials, especially C60 fullerene, have shown promise in the development of highly sensitive and selective sensors. Building on this background, the present study aimed to design and theoretically evaluate a pristine C60-based sensor and its doping forms with B, N, Si for acetone detection using density functional theory (DFT) and quantum theory of atoms in molecules (QTAIM). Key parameters including adsorption energy (Eads), recovery time (τ), electrical conductivity (σ), HOMO–LUMO gap (HLG), and dipole moment (μ) were computationally studied. The results show that SiC59 acts as a highly sensitive sensor, exhibiting a strong adsorption energy of − 137.17 kJ mol^−1^, a reduced HLG of 0.74 eV, a high dipole moment of 19.55 D, and a fast recovery time of 1.50 × 10–10 s. In contrast, BC59 exhibits exceptional adsorption capacity (Eads = − 109.28 kJ mol^−1^), making it ideal for acetone adsorption and environmental remediation. The superior performance of SiC59 and BC59 holds promise for efficient acetone detection and removal, supported by strong quantum mechanical insights.

## Introduction

Heart failure refers to a chronic, progressive clinical syndrome characterized by the inability of the heart to pump sufficient quantity of blood to satisfy the metabolic needs of the body. Heart failure is seen as a global public health problem accompanied by increased morbidity, mortality, and the cost of healthcare. The onset of the disease is gradual, often beginning with structural or functional defects of the heart induced by, for example, hypertension, coronary artery disease, or other cardiomyopathy. If inadequately treated or diagnosed at a late stage, heart failure will unequivocally lead to a decline in quality of life and contribute to heightened risks of hospitalization and death^1,2^.

Therefore, there is a need for early diagnosis of heart failure to allow timely intervention to delay progression of the disease, thereby improving patient outcomes and reducing the economic burden on healthcare. However, the currently employed, most common methods used to diagnose heart failure often entail using echocardiography, electrocardiography, chest X-ray, and measurement of natriuretic peptides. Such diagnostic approaches have significant limitations in the sense that they require specialized equipment and trained personnel, which can be a challenge in resource-poor regions. In addition, most of these tools detect the disease after cardiac dysfunction develops and progresses; hence, their ability to make early diagnoses is limited^3,4^.

Recently, there has been a surge of interest in employing disease biomarkers as a novel approach to identify heart failure early in a patient’s disease. Disease biomarkers are non-invasive, quick, and potentially less expensive options for diagnosis earlier in a disease’s course, prior to major clinical signs and symptoms. In this context, FG Marcondes-Braga et al. have recently identified acetone in exhaled breath as a potentially valuable biomarker for identifying individuals at risk for heart failure^5^. Higher than normal levels of acetone identified in a patient’s breath are correlated with changes in metabolism in patients with heart failure, and may serve as a useful measure of the presence of the disease.

In addition to its importance in medicine, the detection of acetone constitutes another interest at the environmental level. Acetone is a volatile organic compound (VOC) which, if introduced into the environment, may result in water contamination, ecological community disruption, and human health risks associated with chronic exposure^6–9^. Recognizing this dual importance, EH Khader et al. have discussed acetone as an environmental pollutant in industrial waste water and finally the need for monitoring and management of acetone^9^. Therefore, within the importance of acetone in human health and the environment, adapting acetone as a disease biomarker and improving detection of acetone, can allow for two improvements, one improving early diagnosis of disease, and the other addressing a public health concern in the environment.

Non-invasive detection of disease biomarkers in exhaled breath using electrochemical and colorimetric sensors has rapidly emerged as an innovative new paradigm in modern diagnostics. These detection platforms offer excellent new capabilities in terms of non-invasiveness, rapidity, and costs^10–12^. An excellent example of this approach appears in the work of A. A. Khan et al. in developing N- and P-doped C_60_ fullerene sensors for nitric oxide (NO), a critical (and valuable) biomarker for respiratory inflammation in patients with COVID-19^13^. In another example, J. A. Rather et al. demonstrated an exciting advance in diagnosing neurodegenerative diseases with a fullerene (C_60_) graphene oxide composite for homovanillic acid (HVA) detection^14^. Similarly building on these observations, advances in the development of efficient electrochemical sensor and adsorbent designs using nanomaterials, especially carbon-based nanomaterials such as graphene, carbon nanotubes, and fullerenes, are creating a new pathway to detecting (removing) biomarkers (pollutant)^15^. These nanomaterials have large surface areas, excellent electrical conductivity, and excellent chemical stability, making them ideal candidates as sensitive, selective detection platforms.

Of the materials highlighted, fullerene C_60_ has emerged as an attractive material for use in electrochemical and colorimetric sensors. C_60_’s unique spherical structure, electronic properties, and ease of chemical modification, make it appropriate for detecting small biomolecules^16–18^. Several studies have shown the utility of C_60_ as an electrochemical sensor and adsorbent of environmental pollutants in the laboratory setting. For example, K. Y. Tajuddin et al. demonstrated the ability of C_60_-based sensors to visually detect isobutyric acid, a volatile biomarker associated with COVID-19^19^. In another example, J. A. Rather et al. reported fullerene-C_60_ to be one of the most effective adsorbents and sensors for sensitive detection and purification of bisphenol-A (BPA), an endocrine-disrupting chemical often found in plastics^20^. This shows that C_60_ is a multifunctional material that can be applied to environmental and public health-related monitoring.

This study aims to design and propose a new fullerene C_60_-based sensor/adsorbent (and its variants with boron (BC_59_), nitrogen (NC_59_), and silicon (SiC59) doping) for effective acetone sensing, due to acetone being a biomarker for heart failure and an environmental contaminant, as well as the effectiveness of fullerene C_60_ as an electron donor or acceptor in sensors and as adsorbents.

Boron, nitrogen, and silicon were chosen as dopants because they all enhance C_60_’s electronic structure. Boron introduces p-type character, nitrogen introduces n-type character, and silicon prepares the framework/influence the framework and charge distribution, all of which enhance reactivity and detection ability.

Studies indicate that this as-doping enhances C_60_ sensing efficacy. T. Yadav et al. found that B-doped and Si-doped C_60_ enhanced the detection of epinephrine, and M. D. Esrafili et al. found that B- and N-doped C_60_ enhanced detection of adsorption for NO and NO_2_^21,22^. These findings show that B, N and Si doping are important strategies for enhancing fullerene C_60_ (or its derivatives) as a sensing platform.

We present a multifunctional platform that utilizes the unique structural and electronic properties of C_60_ to fulfill both medical diagnostic requirements as well as meet environmental monitoring challenges. To further optimize the design and predict the sensing behavior of the proposed sensor before its experimental synthesis, computational chemistry was utilized as a straightforward and inexpensive avenue. This predictive approach can simplify the development process considerably and facilitate better experimental efforts^23–25^. The interaction of acetone molecules with modified fullerene structures was investigated through DFT and QTAIM in this study^26,27^. The influence of boron, nitrogen, and silicon doping in sensing properties in fullerene C_60_ was specifically investigated. These were chosen from literature evidence whereby it has been shown that boron, nitrogen, and silicon doping enhances the electronic properties and chemical reactivity of C_60_; therefore, making it a better candidate for sensing material^28–30^.

The findings of this study can provide valuable theoretical contributions towards advancing the development of practical and low-cost sensor/sorbents for the early detection of heart failure and for environmental monitoring. We anticipate that such findings may open up further experimental studies and assist researchers with developing the synthesis of new, effective and low-cost sensor/sorbents based on C_60_ fullerenes. This may ultimately lead to effective management for early diagnosis of heart failure and promote enhanced environmental monitoring and control of acetone-based environmental pollutants.

## Computational details

**Fig. 1:**
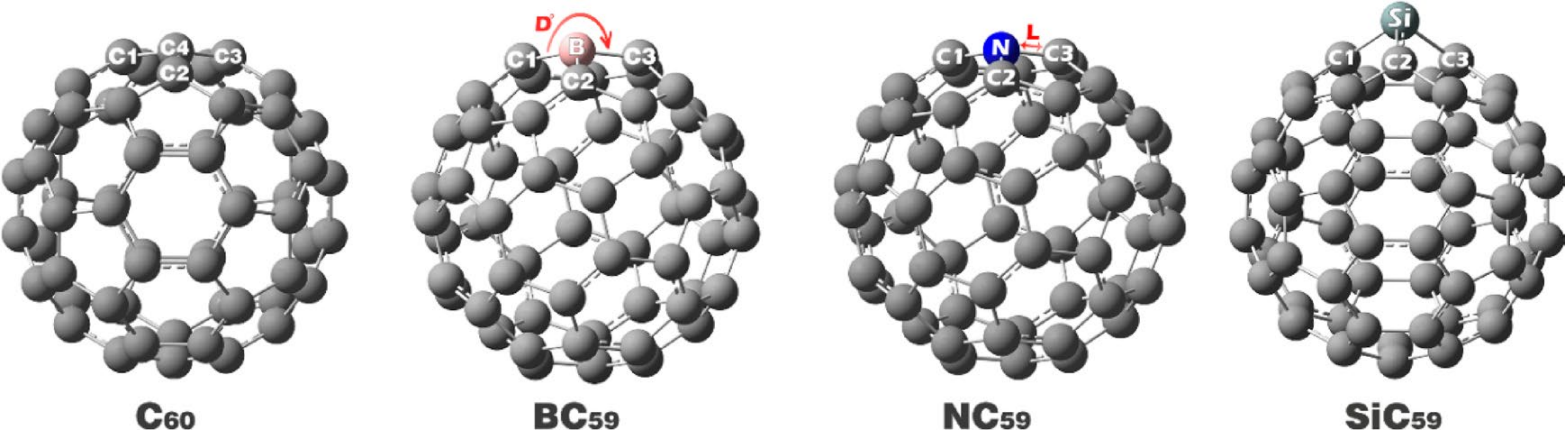
Optimized geometry of pristine C_60_ and its doping forms with boron (BC_59_), nitrogen (NC_59_), and silicon (SiC_59_).

The structural models of C_60_ fullerene nanocage, and the boron doped (BC_59_), nitrogen doped (NC_59_), and silicon doped (SiC_59_) fullerene, as well as acetone and acetone complex, were created using the GaussView 6.0 program (Fig. 1). The geometries of these structures were optimized using Gaussian 09W^31^. The optimization of the structures was done through density functional theory (DFT) with the B97D/ 6-31G(d) computational method. The B97D functional was used for the study because it has an empirical dispersion correction method which is important for accurately representing non-covalent interactions (like van der Waals forces) specific to fullerene-based systems and molecular complex interactions^32,33^. The B97D functional is known to predict the HOMO–LUMO energy gap for C60 fullerene accurately and was really close to experimental data^34^. This also supports the B97D functional option in study.

All calculations while considering solvation effects were done in the water phase with the Conductor-like Polarizable Continuum Model (CPCM)^35^. Water is used as the phase in this simulation to accurately capture the physiological and environmental conditions under which acetone is found. Exhaled air by the human body contains moisture, and acetone directly interacts with water molecules in the respiratory tract when exhaled, which may affect its ability as a biomarker tethered to the water biomarker. Environmentally acetone is highly soluble in water, and water may contain acetone or other organic pollutants that impact aquatic systems. Water, as a phase, offers a realistic modeling environment for acetone’s solubility, diffusion, and interaction model used, supporting the overall validity of this work and application for use as a biomarker and knowledge of its behavior in a specific phase and out in the environment as a contaminant.

Additionally, frequency calculations were performed at the same theoretical level to ensure that the optimized structures are stable. No imaginary frequencies in the vibrational spectra indicated the geometries obtained corresponded to real energy minima instead of transition states.

To assess the structural stability of C_60_ fullerene nanocages and their doped composed materials (BC_59_, NC_59_, SiC_59_), the cohesive energy was obtained via Eq. 1 (All cohesive energy values were calculated in terms of kJ mol^−1^). This measure indicates how strongly atoms are bound to one another within the system and therefore the stability of the pristine structures compared to the doped ones^36^.

1 $$E_{Coh} = \frac{{ - \left( {E_{{{\text{Complex}}}} - \sum E_{{{\text{individual}}}} } \right)}}{n}$$

E_Complex_ = Total energy of the full structure (e.g., C_60_ or doped C_60_). ∑E_Individual_ = Sum of energies of isolated atoms/fragments. n = Number of atoms in the system.

The electronic and quantum properties (including the HOMO–LUMO energy gap (HLG), chemical hardness (η), chemical potential (µ), and chemical softness (S)) were calculated for both pristine and complexed structures using Eqs. 2–5 (HLG, η, and µ values were reported in eV, and S values were reported as eV^−1^). These parameters were computed to quantify how structural modifications (doping and complexation) influence the systems’ reactivity, stability, and charge transfer behavior^37,38^.

2 $${\text{HLG}} = \left| {{\text{E}}_{{{\text{HOMO}}}} - {\text{E}}_{{{\text{LUMO}}}} } \right|$$

3 $${\upeta } = \frac{{\left( { - {\text{E}}_{{{\text{HOMO}}}} - \left( { - {\text{E}}_{{{\text{LUMO}}}} } \right)} \right)}}{2}$$

4 $${\upmu } = \frac{{ - \left( { - {\text{E}}_{{{\text{HOMO}}}} + \left( { - {\text{E}}_{{{\text{LUMO}}}} } \right)} \right)}}{2}$$

5 $${\text{S}} = \frac{1}{2}{\upeta }$$

In these calculations, E_HOMO_ denotes the energy of the Highest Occupied Molecular Orbital (HOMO), while E_LUMO_ represents the energy of the Lowest Unoccupied Molecular Orbital (LUMO).

The maximum charge transfer (ΔNmax) and electrophilicity-based charge transfer (ECT) were calculated using Eqs. 6 and 7, where (ΔN_max_)_α_ and (ΔN_max_)_β_ represent the maximum charge transfer values for (R-)C60 (species α) and acetone (species β), respectively^39,40^.

6 $$\Delta N_{\max } = - \frac{\mu }{\eta }$$

7 $$ECT = \left( {\Delta N_{\max } } \right)_{\alpha } - \left( {\Delta N_{\max } } \right)_{\beta }$$

The ECT parameter provides critical insight into the direction of charge transfer between these systems. When ECT > 0, it indicates that C_60_ possesses a greater electron-accepting capability than acetone, resulting in net electron transfer from acetone to the C_60_ nanocage. Conversely, when ECT < 0, acetone demonstrates superior electron-accepting ability compared to C_60_, leading to charge transfer in the opposite direction from C60 to acetone^41^.

The adsorption characteristics and electronic response of the C_60_-acetone system were quantitatively evaluated through three key parameters calculated using Eqs. 8–10. The adsorption energy (Eads) was determined through Eq. 8, which accounts for the energy difference between the complexed system (E_Complex_) and its isolated components (E_Acetone_ and E_(R-)C60_), while incorporating the Basis set superposition error (E_BSSE_) correction for enhanced accuracy. The Eads values were calculated and reported in terms of kJ.mol^−1^^42^.

8 $$E_{ads} = E_{Complex} - \left( {E_{{{\text{Acetone}}}} + E_{{\left( {R - } \right)C60}} } \right) + E_{BSSE}$$

The recovery time (τ), calculated via Eq. 9, describes the thermal desorption kinetics of acetone from the C_60_ surface, where v_0_ represents the attempt frequency (10^12^ s^−1^), T the temperature (298K), and k_B_ denotes the Boltzmann constant^43^. τ values were reported in seconds (s).

9 $$\tau = V_{0}^{ - 1} \times \exp \left( { - \frac{{E_{ads} }}{{k_{B} T}}} \right)$$

Finally, Eq. 10 was employed to compute the electrical conductivity (σ), which exhibits an exponential dependence on both temperature (T) (298 K) and the energy gap (HLG), with A representing the Richardson constant (6 × 10^5^ A m^−2^) and K being the Boltzmann constant^44^. The σ values were reported in A.m^−2^.

10 $$\sigma = AT^{3/2} e^{{\left( { - HLG/2KT} \right)}}$$

Also, the dipole moment (µ) (In terms of Debye units (D)) for each of the studied structures was calculated using Eq. 11^45^.

11 $${\upmu } = \sqrt {{\upmu }_{{\text{x}}}^{2} + {\upmu }_{{\text{y}}}^{2} + {\upmu }_{{\text{z}}}^{2} }$$

This computational approach ensures reliable predictions of structural and electronic properties for the studied systems, providing a solid foundation for further analysis.

## Results and discussion

### Structural properties

#### Bond length and bond angle

The analysis of bond lengths (L) and bond angles (D) is a key criterion in molecular design, and there is potential to vary or engineer systems through doping and functionalization since these structural parameters inherently influence the electronic, mechanical, and chemical properties of the resulting material. In the case of C60 fullerene and its doped variants (B, N, Si), even small changes of bond length (due to heteroatom inclusion) can induce substantial changes to the π-conjugation and strain distribution within the carbon cage that in turn alter its conductivity, chemical reactivity, and stability^46^. Based on this, the bond lengths and angles were calculated for each of the designed structures, and the results are presented in Table 1.

**Table 1:** Bond length (A) and bond angle (Dᵒ) of some important atoms in each of the studied structures (Bond length values are in Angstroms (Å) and bond angle values are in degrees (°)).

Structure	Bond lengths (Å)		Bond angles (°)	
C_60_	C1-C4	1.45	C1-C4-C2	119.9
	C2-C4	1.40	C1-C4-C3	107.9
	C3-C4	1.45	C2-C4-C3	119.9
BC_59_	C1-B	1.53	C1-B-C2	118.4
	C2-B	1.55	C1-B-C3	118.4
	C3-B	1.55	C2-B-C3	106.2
NC_59_	C1-N	1.40	C1-N-C2	119.2
	C2-N	1.42	C1-N-C3	119.2
	C3-N	1.42	C2-N-C3	107.4
SiC_59_	C1-Si	1.86	C1-Si-C2	99.4
	C2-Si	1.81	C1-Si-C3	90.3
	C3-Si	1.86	C2-Si-C3	99.4

In pristine C_60_, the C–C distances around the probed site alternate between 1.40 and 1.45 Å, and the angles cluster near the ideal sp^2^ values of ~ 120° with the characteristic ~ 108° pentagonal angle, so the π system is broadly delocalized over a nearly uniform network of p orbitals. Once a heteroatom replaces a carbon, the local geometry departs from this pattern, and the π electrons respond by flowing away from regions where p–p overlap is weakened and toward areas where conjugation can still be efficiently sustained. In BC_59_, the B–C bonds elongate to 1.53–1.55 Å and the B-centered angles open to 118.4°/118.4° with a compressed 106.2° between the two adjacent carbons, reflecting the electron deficiency of boron and a slight reduction in effective π overlap on the B site. The π density therefore migrates from the B vicinity onto the neighboring carbon atoms of the cage, which act as reservoirs to compensate for the local withdrawal, producing a polarized π framework with partial positive character on B and enhanced π electron density on the adjacent carbons. In NC_59_, the N–C bonds are comparatively short (1.40–1.42 Å) and the angles (119.2°/119.2°/107.4°) remain close to the undoped values, consistent with nitrogen injecting electron density into the cage. Here, the π electrons are partially pushed from the N lone-pair/π manifold into the surrounding conjugated network. Still, because the geometry retains near-sp^2^ metrics, the additional charge is more smoothly redistributed rather than sharply localized, yielding a locally enriched yet still delocalized π region around the dopant. In SiC_59_, the Si–C bonds expand markedly to 1.81–1.86 Å and the angles collapse to 99.4°/90.3°/99.4°, which strongly perturbs the planarity and orthogonality required for efficient π overlap. The silicon site thus behaves as a π “break,” and the π electrons retreat from the distorted, quasi-sp^3^-like environment around Si toward the undistorted carbon framework, concentrating the delocalized π cloud on the opposite side of the defect and along paths that preserve C–C p-orbital alignment.

#### Cohesive energy

**Fig. 2:**
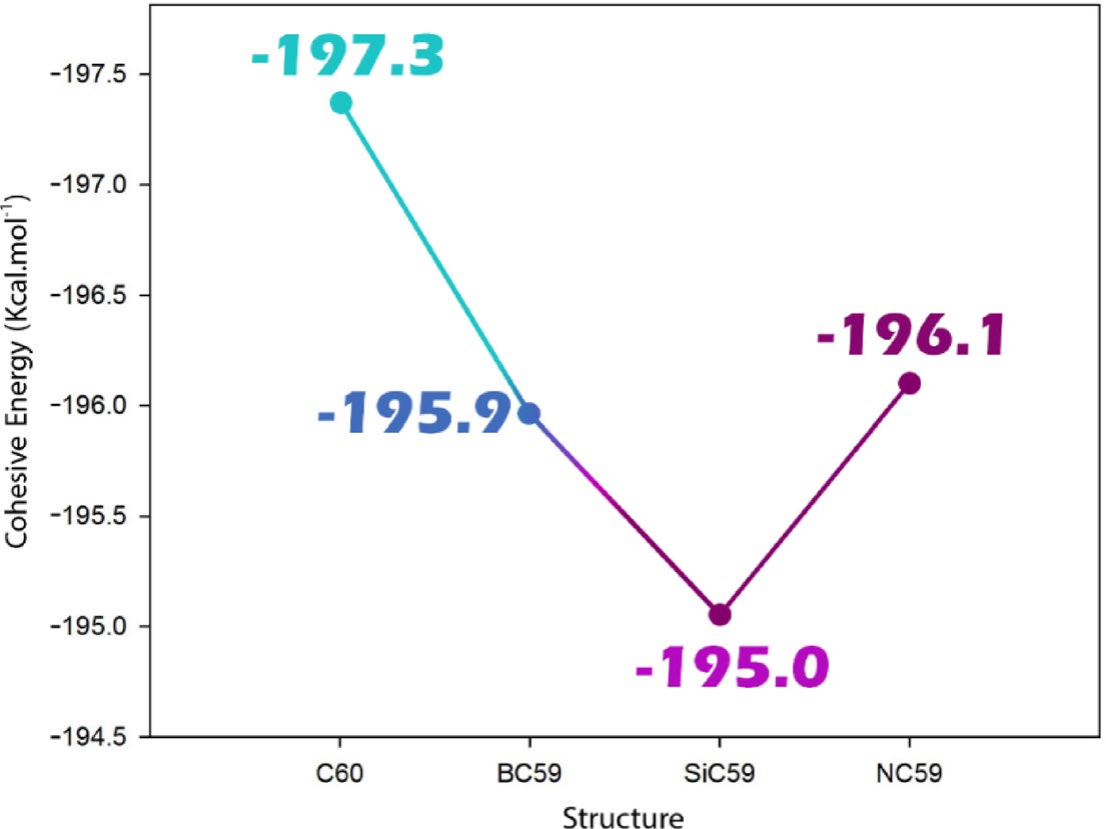
Cohesive energy for pristine C_60_ and its doped forms with B, N, and Si.

Cohesive energy can be understood as the energy needed to break down a crystalline or molecular material into its individual atoms in the gaseous phase, and it acts as a first order indicator of structural stability and bonding strength. It is important to consider the coherence energy after doping because the addition of heteroatoms can weaken or strengthen the structure overall. In sensor design, having a more negative cohesive energy can be beneficial for mechanical stability and resistance to structural failure during the adsorption–desorption stage. In addition, the changes in cohesive energy give insight into how the local electronic density and π-conjugation are altered in the system, which gives a direct influence on the potential sensitivity and selectivity of the sensor to target analytes^47^. Accordingly, the coherent energy was calculated for each of the studied structures, and the results were reported in Fig. 2.

The cohesive energy of pristine C_60_ ( −197 kJ mol^−1^) drops only marginally upon single-atom substitution: to −196  kJ mol^−1^ for NC_59_ and to −195  kJ mol^−1^ for both BC_59_ and SiC_59_. These ≤ 2  kJ mol^−1^ (≈1%) reductions indicate that all doped cages remain thermodynamically robust, i.e., the global bonding network is only slightly weakened despite the local geometric and electronic distortions introduced by the dopants. The smallest change for NC_59_ is consistent with nitrogen’s ability to supply electron density and form relatively strong, near‑sp^2^ C–N bonds, thereby perturbing the lattice least. In contrast, the comparable decreases for BC_59_ and SiC_59_ reflect, respectively, boron’s electron deficiency and silicon’s size/hybridization mismatch, both of which modestly diminish average bond strengths. From a sensing perspective, such minor cohesive-energy penalties imply that the doped fullerenes should preserve mechanical/chemical integrity during adsorption–desorption cycles, while the dopant-induced redistribution of π electrons (rather than any large loss of cohesion) will be the dominant lever for tuning sensitivity and selectivity. The trends in cohesive energy changes confirm that the contamination disrupts the host framework, but does not destabilize it, confirming these structures as suitable sensing platforms.

#### MEP contours

**Fig. 3:**
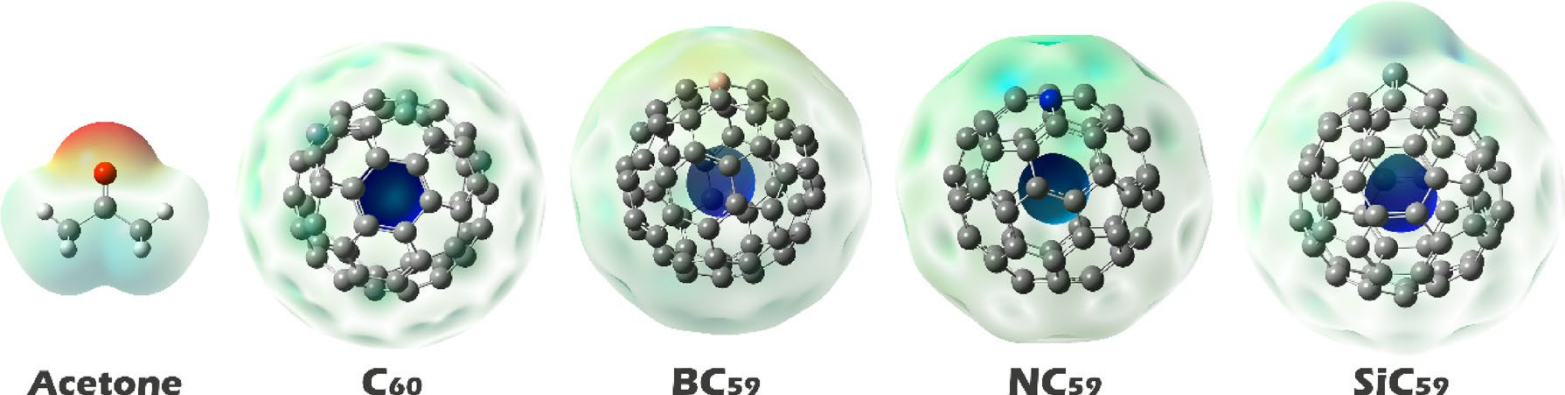
MEP contours for acetone, C_60_, and its doping forms.

Molecular Electrostatic Potential (MEP) contours are crucial for determining the most reactive sites on a molecule because they illustrate the electrostatic potential surface, which illustrates the distribution of the electrostatic potential due to the nuclei and electron cloud. Regions with negative potential (electron-rich) represent where electrophilic attacks are most likely to occur, while regions with positive potential (electron deficient) favor nucleophilic attacks. The coloring of these contours goes from red to blue; red indicates regions of high electron density (strong negative potential), blue represents regions with positive potential (electron deficient), and yellow to green represents neutral or less reactive potential. By examining MEP contours, one can predict molecular interactions, sites of adsorption, or analyte-binding in sensors. MEP contour maps of all of the designed structures are shown in Fig. 3^48^.

**Fig. 4:**
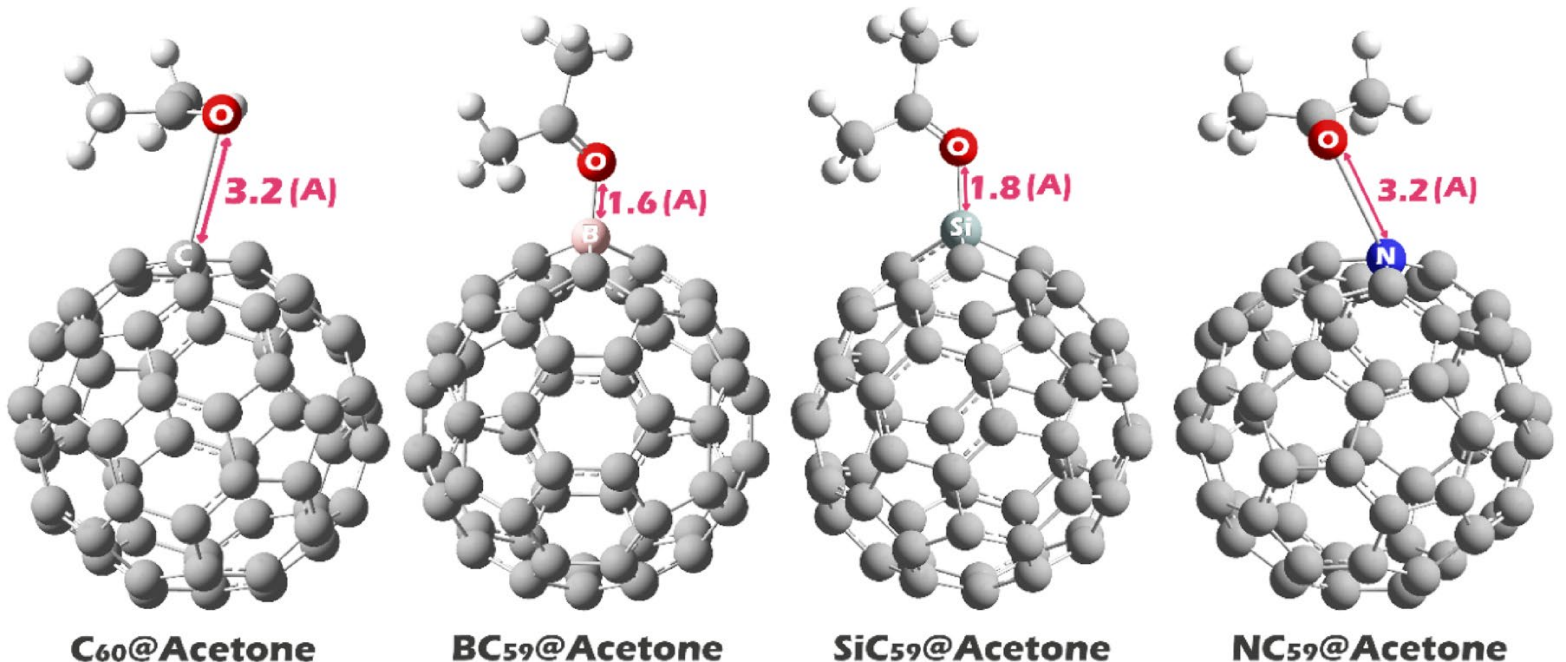
The optimized geometric structure of each of the complexes designed in this work.

In Molecular Electrostatic Potential (MEP) maps, acetone displays a pronounced red lobe centered on the carbonyl oxygen, signifying a region of maximum negative potential and therefore the primary nucleophilic (electron‑donor) site; accordingly, this oxygen will seek the most electropositive (blue) regions on any partner surface to minimize the electrostatic component of the interaction energy and to enable n(O) → acceptor orbital donation. In pristine C_60_ the blue potential is located on the carbon atoms, reflecting a slight electron deficiency of the curved π surface; the acetone oxygen is thus expected to approach one (or a small patch) of cage carbons, forming a predominantly electrostatic/dispersion‑assisted charge‑transfer complex in which the oxygen lone pair donates weakly into low‑lying π* states of the fullerene. In BC_59_ the blue region localizes on the boron atom, consistent with boron’s electron deficiency and the availability of an empty p orbital; here the interaction should be stronger and more directional, with appreciable n(O) → p(B) donation and a correspondingly larger charge transfer, making the B site the preferred anchoring point for acetone (see Fig. 3). In NC_59_ the MEP indicates that the nitrogen atom is the most positive site, implying that the dopant has pulled electron density away from itself (e.g., due to the global redistribution within the cage), so the acetone oxygen will preferentially coordinate to N, again through lone‑pair donation into N‑centered acceptor orbitals; however, because N typically possesses a filled valence shell, the interaction may be less strongly covalent than in the boron case and more governed by electrostatics/polarization. In SiC_59_ the blue potential accumulates on silicon, whose low electronegativity and larger, more polarizable valence shell produce a strongly electrophilic locus; the oxygen lone pair can engage in a dative O → Si bond with partial covalent character (n(O) → σ*(Si–C)), likely accompanied by noticeable charge transfer and local geometric relaxation. According to the MEP patterns, acetone is predicted to complex at the carbon sites on pristine C_60_, at the dopant atom itself in all heteroatom-substituted cages (B in BC_59_, N in NC_59_, Si in SiC_59_), and the strength of the interaction and the degree of donor–acceptor charge transfer should be maximum at the centers that are strongly blue (strongly electrophilic) and inherently electron-deficient (especially B and Si), with the primary C_60_ contact being the weakest. Based on this, each of the complexes was designed and their geometric structure was optimized (see Fig. 4).

In the designed complexes, the X–O bond length follows the order B–O (1.6 Å) < Si–O (1.8 Å) ≪ C–O ≈ N–O (3.2 Å). The B–O and Si–O values lie close to the sums of the tabulated covalent radii for the respective atom pairs (B + O ≈ 1.50 Å, Si + O ≈ 1.77 Å), whereas the C–O and N–O separations exceed the analogous sums for C–O (≈1.42 Å) and N–O (≈1.37 Å) by nearly 2 Å. This contrast shows that B and Si substitution leads to geometries in which the oxygen atom is positioned much nearer to the endohedral heteroatom, while in the pristine (C) and N-doped cages, the optimized structures maintain a far larger X–O distance (~ 3.2 Å). The pattern can be attributed to differences in atomic size and electronic distribution introduced by the heteroatoms.

### Electronic properties

#### Frontier orbital energy and quantum parameters

In electrochemical sensor design, the HOMO–LUMO energy gap (HLG) is crucial as it reflects the electronic excitability and charge-transfer capability of the material; a smaller HLG often enhances sensitivity by facilitating electron transfer during analyte adsorption. Chemical hardness (η), defined as half of the HLG, indicates the resistance of the sensor material to charge deformation, with lower hardness favoring higher reactivity toward target molecules. Chemical potential (µ) describes the tendency of electrons to escape from the material, directly influencing the interaction strength with analytes and the sensor’s electronic response. Chemical softness (S), the inverse of hardness, represents the material’s ability to adapt its electronic structure under external perturbation, which is vital for detecting subtle molecular interactions^49,50^. Together, these quantum parameters determine the sensor’s reactivity, stability, and efficiency in electron exchange, directly impacting its performance. Therefore, each of these parameters was subjected to a computational study, and the results were reported in Table 2.

**Table 2:** Calculated values of HOMO/LUMO orbital energies, energy gap (HLG), chemical hardness (η), chemical softness (S), chemical potential (μ), Maximum electronic charge (ΔNmax), and Electrophilicity-Based Charge Transfer Descriptor (ECT) for the studied compounds.

Structure	**LUMO (eV)**	**HOMO (eV)**	**HLG (eV)**	**η (eV)**	**μ (eV)**	**S (eV**^**−1**^**)**	${\Delta {\varvec{N}}}_{{\varvec{m}}{\varvec{a}}{\varvec{x}}}$	**ECT**
C60	− 3.7	− 5.38	1.68	0.84	− 4.54	0.59	5.40	–
BC59	− 3.58	− 4.93	1.35	0.67	− 4.25	0.74	6.30	–
NC59	− 3.79	− 5.2	1.41	0.70	− 4.49	0.70	6.37	–
SiC59	− 3.94	− 5.12	1.18	0.59	− 4.53	0.84	7.67	–
C60@ Acetone	− 3.78	− 4.43	0.65	0.325	− 4.10	1.53	12.63	− 7.22
BC59@ Acetone	− 3.95	− 4.93	0.98	0.49	− 4.44	1.02	9.06	− 2.75
NC59@ Acetone	− 3.2	− 4.86	1.66	0.83	− 4.03	0.60	4.85	1.52
SiC59@ Acetone	− 3.6	− 4.34	0.74	0.37	− 3.97	1.35	10.72	− 3.05

**Fig. 5:**
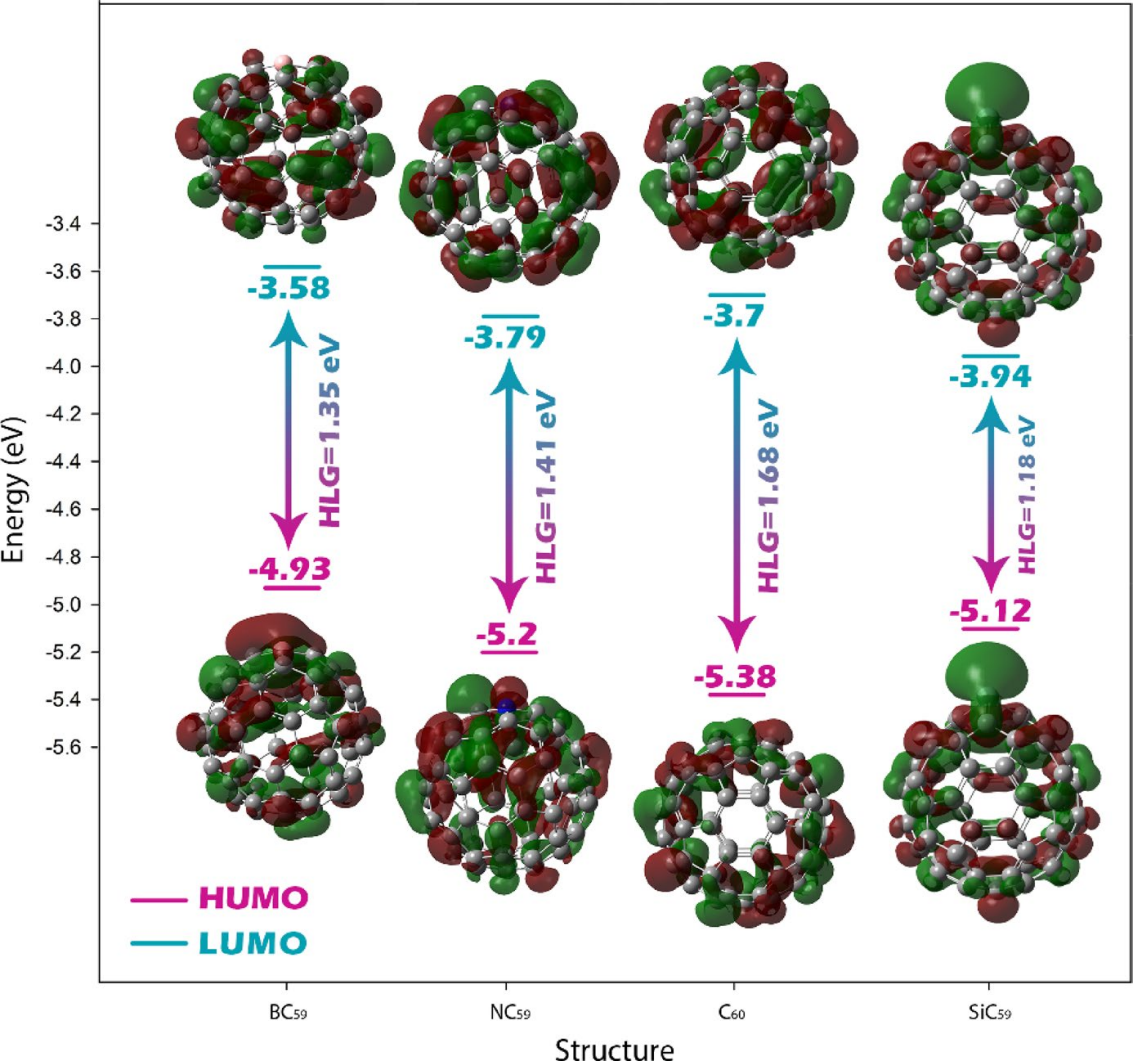
The trend of changes in the energy gap of pristine C_60_ after doping with B, Si, and N.

First, examining the values for C60 before and after doping, it is clear that doping induces significant modifications in the electronic structure. Pure C60 exhibits a HOMO of − 5.38 eV, a LUMO of − 3.70 eV, and a corresponding HOMO–LUMO gap (HLG) of 1.68 eV, along with a chemical hardness (η) of 0.84 and chemical softness (S) of 0.59. Upon doping, all three derivatives show a narrowing of the energy gap, which decreases to 1.35 eV for BC59, 1.41 eV for NC59, and 1.18 eV for SiC59. This gap reduction is accompanied by an increase in softness (up to 0.84 for SiC59), indicating enhanced polarizability and higher electronic reactivity. At the same time, the maximum electronic charge transfer (ΔNmax) rises considerably for the doped systems, especially for SiC59 (7.67 compared to 5.40 for C60), suggesting stronger ability to accept electronic charge. These trends confirm that doping with B, N, or Si makes the fullerene cage more chemically active and potentially more sensitive to analyte interaction (Fig. 5).

Importantly, the energy gap reported for pristine C_60_ (1.68 eV) falls within the same range as the experimental HLG values determined in conductivity-based studies. Kremer et al. and Rabenau et al. reported gaps in the range of 1.6–1.9 eV for C_60_, which strongly supports the accuracy and reliability of the computational method used here.

When examining the interaction with acetone, the data reveal pronounced changes depending on the structure. The pristine C_60_-acetone complex shows a dramatic reduction in the energy gap to 0.65 eV, a steep decrease in hardness (0.325 eV), and a more than two-fold increase in softness (1.53 eV^−1^). Moreover, ΔNmax rises sharply to 12.63. The doped fullerenes also exhibit notable variations, although the trends differ among them. For instance, BC_59_@Acetone maintains a relatively small gap of 0.98 eV and moderate softness (1.02), while NC_59_@Acetone shows a gap close to that of pristine C_60_ (1.66 eV) and the lowest ΔNmax (4.85), indicating weaker interaction. SiC_59_@Acetone again demonstrates strong responsiveness, with a reduced gap of 0.74 eV, high softness (1.35), and elevated ΔNmax (10.72). The negative values of the electrophilicity-based charge transfer descriptor (ECT) for C_60_@Acetone (− 7.22), BC_59_@Acetone (− 2.75), and SiC_59_@Acetone (− 3.05) clearly demonstrate that acetone acts as an electron donor in these complexes. By contrast, the positive ECT value observed for NC_59_@Acetone (+ 1.52) suggests that in this case, acetone behaves more like an acceptor.

Comparing these values, it is evident that C_60_@Acetone and SiC_59_@Acetone exhibit the most pronounced electronic changes upon interaction, with significant gap reduction, enhanced softness, and large charge transfer values. These parameters suggest that these two structures are the most sensitive to the presence of acetone and could serve as effective sensing materials. By contrast, NC_59_@Acetone shows the least response, with minimal alterations in its frontier orbital energies and electronic descriptors, implying limited applicability as an acetone sensor. However, it is essential to emphasize that these findings are based solely on theoretical predictions derived from calculated quantum parameters. While such parameters provide valuable first insights into the electronic behavior and potential sensing/adsorption ability of the studied structures, they represent only an initial step in the evaluation process. In order to reliably determine which structure is the most suitable sensor or absorber for acetone, further analyses are required. These additional evaluations are discussed in the following sections.

**Fig. 6:**
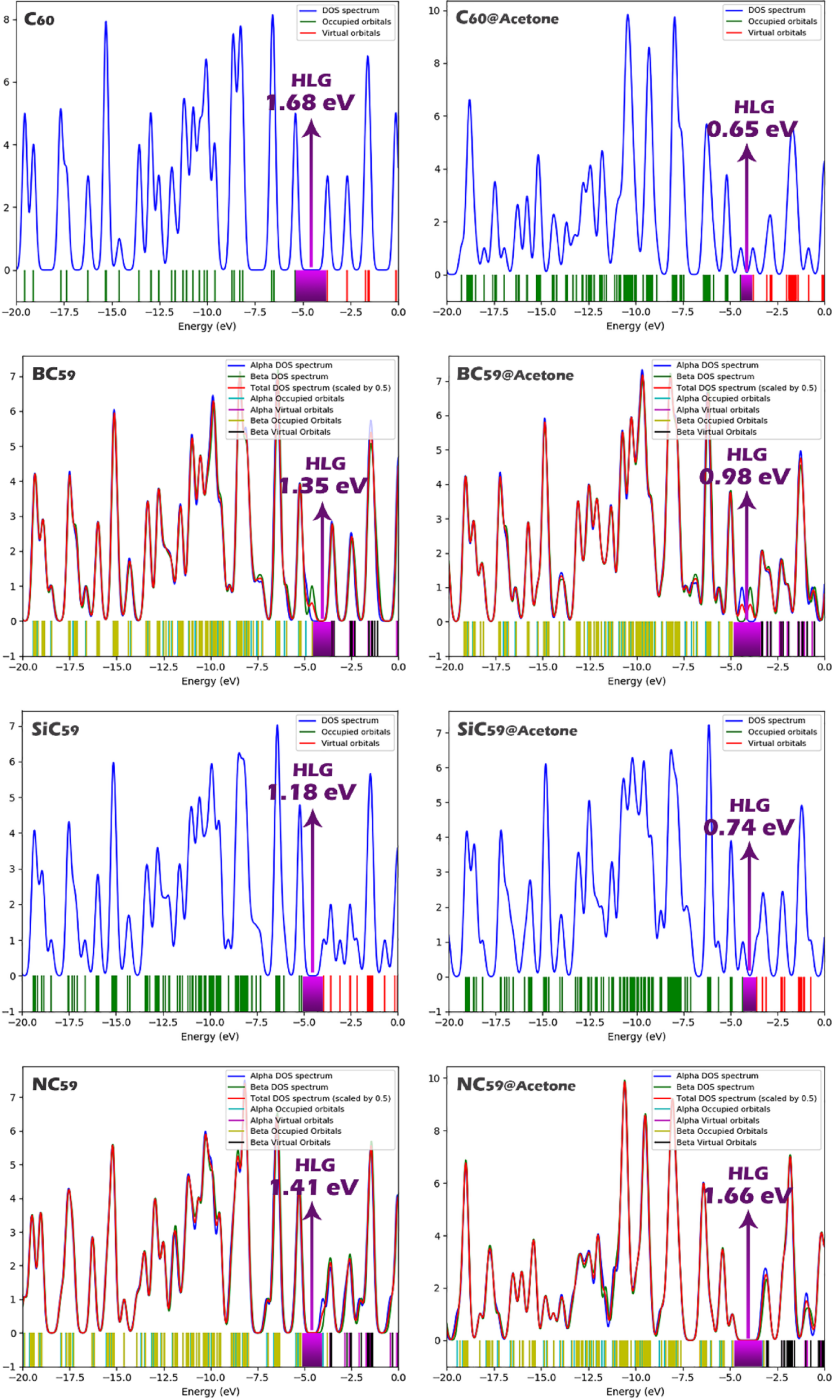
DOS plots for each of the structures studied in this work.

Density of States (DOS) plots provide a graphical representation of the distribution of electronic states over energy levels, allowing direct visualization of the positions of the HOMO and LUMO orbitals and the energy gap between them. In the DOS plots, the energy range where no electronic states are present corresponds to the HLG. By comparing the HOMO and the LUMO, the size of this gap can be accurately determined (See Fig. 6). The DOS plots for C_60_, BC_59_, NC_59_, and SiC_59_, as well as their acetone complexes, clearly illustrate the narrowing or widening of the energy gap reported in Table 2. For example, the DOS plot of SiC_59_ shows the closest proximity of HOMO and LUMO levels, confirming its smallest HLG of 1.18 eV among the undoped and doped structures. Similarly, the pronounced gap reduction in C_60_@acetone (0.65 eV) is clearly visible as a much narrower region without states between the HOMO and LUMO peaks. Thus, the DOS analysis directly validates the calculated HOMO, LUMO, and HLG values in Table 2.

**Fig. 7:**
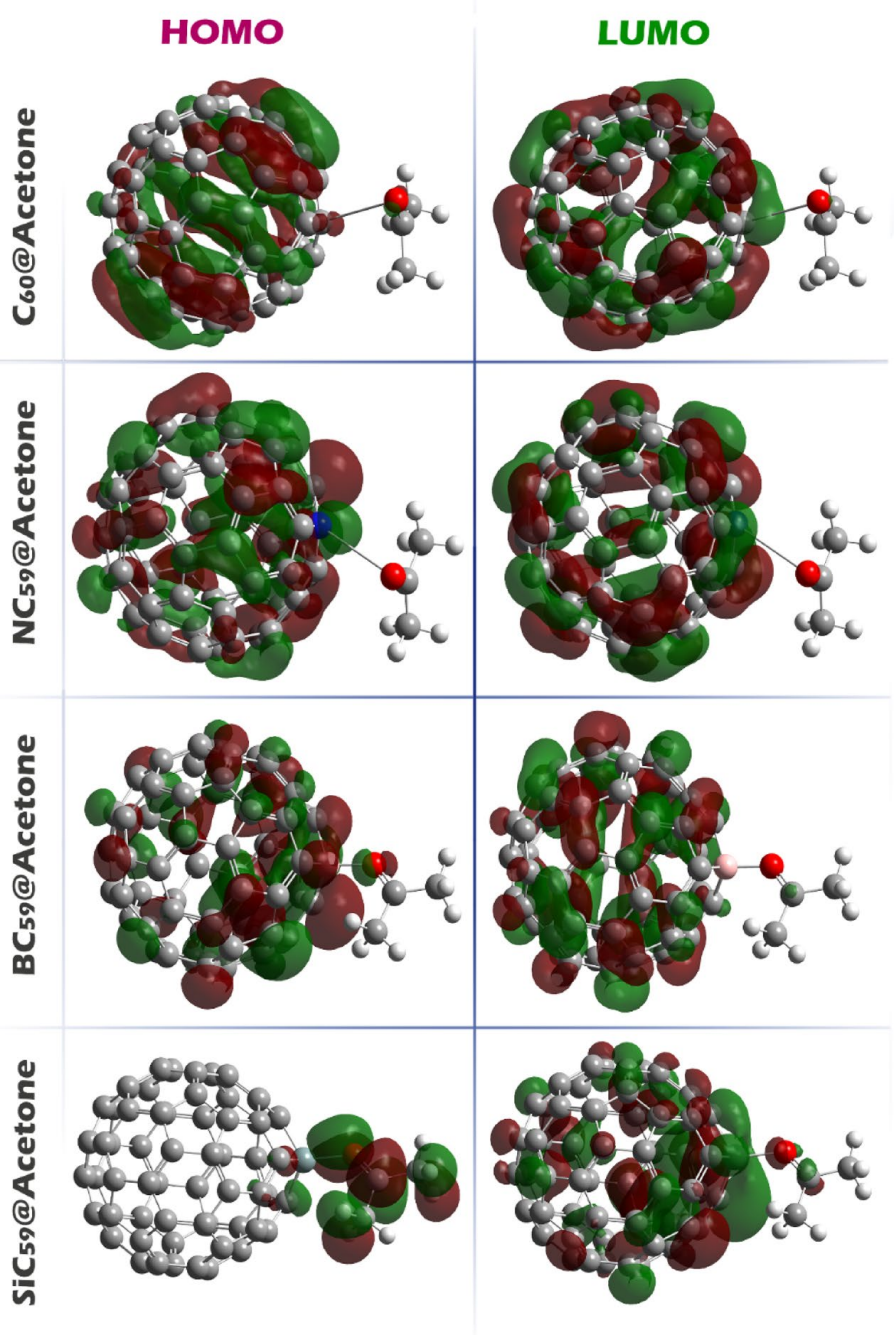
The spatial shape and how the HOMO and LUMO orbitals are distributed in each of the designed complexes.

Assessing the spatial shape and distribution of the HOMO and LUMO orbitals is fundamental because it pinpoints where electrons can be removed from HOMO or accepted into LUMO, thereby dictating the direction, magnitude, and efficiency of charge transfer, the nature of the dominant electronic transitions, and ultimately the sensitivity and selectivity of an electrochemical sensor. When both frontier orbitals reside on the sensor, the analyte mainly perturbs the sensor’s electronic structure without directly participating in the lowest‑energy donor–acceptor channel; the response is then governed by intra‑sensor excitations and relatively weak orbital overlap with the adsorbate. This is precisely what is observed for C_60_@Acetone, NC_59_@Acetone, and BC_59_@Acetone, where localization of both HOMO and LUMO on the sensor implies that acetone acts largely as an external electrostatic/polarizing influence, with limited direct frontier‑orbital mixing (See Fig. 7). In contrast, in SiC_59_@Acetone the HOMO is localized on acetone while the LUMO lies on the sensor, creating an energetically and spatially directed donor → acceptor pathway from the analyte to the sensing material (This result overlaps with the reported negative ECT values for SiC_59_@Acetone). This spatial separation of frontier orbitals maximizes the driving force for analyte‑to‑sensor electron transfer, enhances the probability of charge‑transfer excitations, and should manifest as a stronger modulation of the sensor’s electrochemical signal (e.g., larger current or potential shifts upon adsorption).

#### Dipole moment

**Fig. 8:**
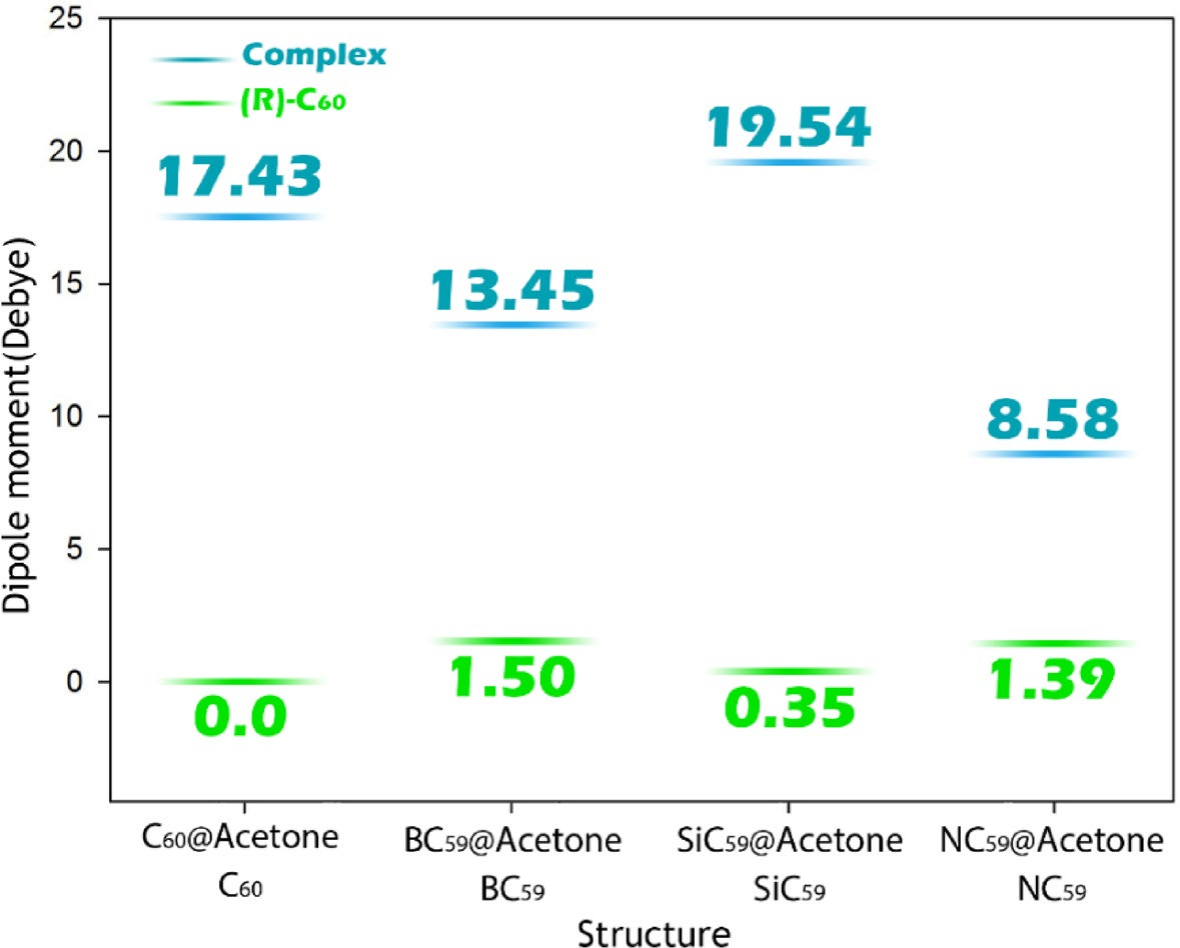
Dipole moment for pristine _C60_ and its doped forms in the presence/absence of acetone.

The dipole moment is an important parameter for the design of electrochemical sensors, since it informs equal polarity of the material affecting salt solubility and electrical signals generation. Typically, the higher the dipole moment, the increased solubility in polar solvents will afford improved dispersion and stability of the sensor material in aqueous or polar media, which is crucial for further applications. Also, by assessing the change in dipole moment by a analyte adsorption, it can reflect charge redistribution and polarization occurring within the sensor-analyte complex, in which this may also lead to a measurable electrical signal in current or potential. Therefore, by taking dipole moments into consideration, it can predict compatibility of the sensor with different media and also be used to gauge strength and direction of charge transfer processes, both of which are essential for the sensor’s sensitivity and selectivity. In this regard, the dipole moment was calculated for each of the designed structures, and the results are shown in Fig. 8.

The dipole moment data reveal significant changes upon acetone adsorption, indicating substantial charge redistribution and polarization within the sensor-analyte complexes. Pristine C_60_, with a dipole moment of 0.0 D due to its perfectly symmetric structure, exhibits a dramatic increase to 17.44 D in the C_60_@Acetone complex. This sharp rise highlights the strong perturbation of the electronic density caused by acetone adsorption, suggesting a pronounced polar interaction and a strong ability to generate detectable electrical signals. In BC_59_, the initial dipole moment of 1.50 D (resulting from the asymmetry introduced by boron doping) increases to 13.45 D upon complexation, reflecting effective charge transfer between the boron site and acetone, which aligns with the short B–O bond length and enhanced electronic coupling observed earlier. SiC_59_ also shows a remarkable increase from 0.35 D to 19.55 D, the highest value among all complexes, indicating intense polarization and the formation of a highly responsive electronic interface between the sensor and acetone. This significant enhancement supports the idea that Si doping not only increases reactivity but also maximizes signal generation due to strong dipole-induced interactions.

In contrast, NC_59_ starts with a dipole moment of 1.39 D but rises only to 8.58 D after adsorption, a relatively small increase compared to the other doped systems. This moderate change correlates with the longer N–O distance and weaker interaction of acetone with the nitrogen site, resulting in lower charge redistribution and potentially weaker electrical signal generation. Overall, the data indicate that acetone adsorption significantly amplifies the polarity of all sensors, with SiC_59_ and C_60_ showing the largest dipole moment changes, implying the highest potential for strong and easily measurable electrochemical responses, while NC_59_ exhibits the weakest polarization effect among the studied complexes.

### Sensor mechanism

#### Adsorption energy, recovery time, and electrical conductivity

Recovery time (τ), electrical conductivity (σ), and adsorption energy (Eads) serve as important metrics to assess the sensor’s mechanism and performance. Adsorption energy (Eads) is associated with the strength of interaction between a sensor and analyte, an Eads that is too weak may lead to poor adsorption exiting the target analyte from the surface, however too strong of an Eads may lead to difficult desorption and to the sensor being unable to regenerate properly to detect additional analyte. Recovery time reflects the time it takes for the sensor to return to its initial state after the analyte has been removed from the surface, which is very important for assessments of reusability, and for functionality in line with monitoring capabilities in real-time systems. Electrical conductivity σ directly Davis a sensors level of sensitivity and its signal output. As the analyte interacts with the sensor surface, electrical conductivity will change, signaling the presence of the adsorbed analyte. When reviewed, these three parameters provide a full understanding of how well a sensor interacts with target molecules and adsorbs to its surface, how long it takes to return to its original state, and how efficiently that interaction is transformed into quantifiable electrical signals.

The data presented in Table 3 provide key insights into the performance of the studied sensor systems for the detection of acetone, a well-known biomarker for non-invasive diagnosis of heart failure. Among the undoped structures, all show relatively similar baseline electrical conductivities (σ) in the range of 2.19 × 10^9^ to 2.43 × 10^9^ A m^−2^, with SiC_59_ having the highest intrinsic conductivity, suggesting it offers a more favorable electronic platform even before interaction with acetone.

**Table 3:** Electrical conductivity (σ), adsorption energy (Eads) and recovery time (τ) values in each of the studied structures.

Structure	Eads (kJ.mol^−1^)	τ (s)	(σ) (A.m^−2^)
C_60_	–	–	$2.19\times {10}^{9}$
BC_59_	–	–	$2.35\times {10}^{9}$
NC_59_	–	–	$2.32\times {10}^{9}$
SiC_59_	–	–	$2.43\times {10}^{9}$
C_60_@Acetone	− 35.81	$3.92\times {10}^{-11}$	$2.70\times {10}^{9}$
BC_59_@ Acetone	− 109.28	$1.19\times {10}^{-10}$	$2.53\times {10}^{9}$
NC_59_@ Acetone	− 39.67	$4.35\times {10}^{-11}$	$2.20\times {10}^{9}$
SiC_59_@ Acetone	− 137.17	$1.50\times {10}^{-10}$	$2.65\times {10}^{9}$

**Fig. 9:**
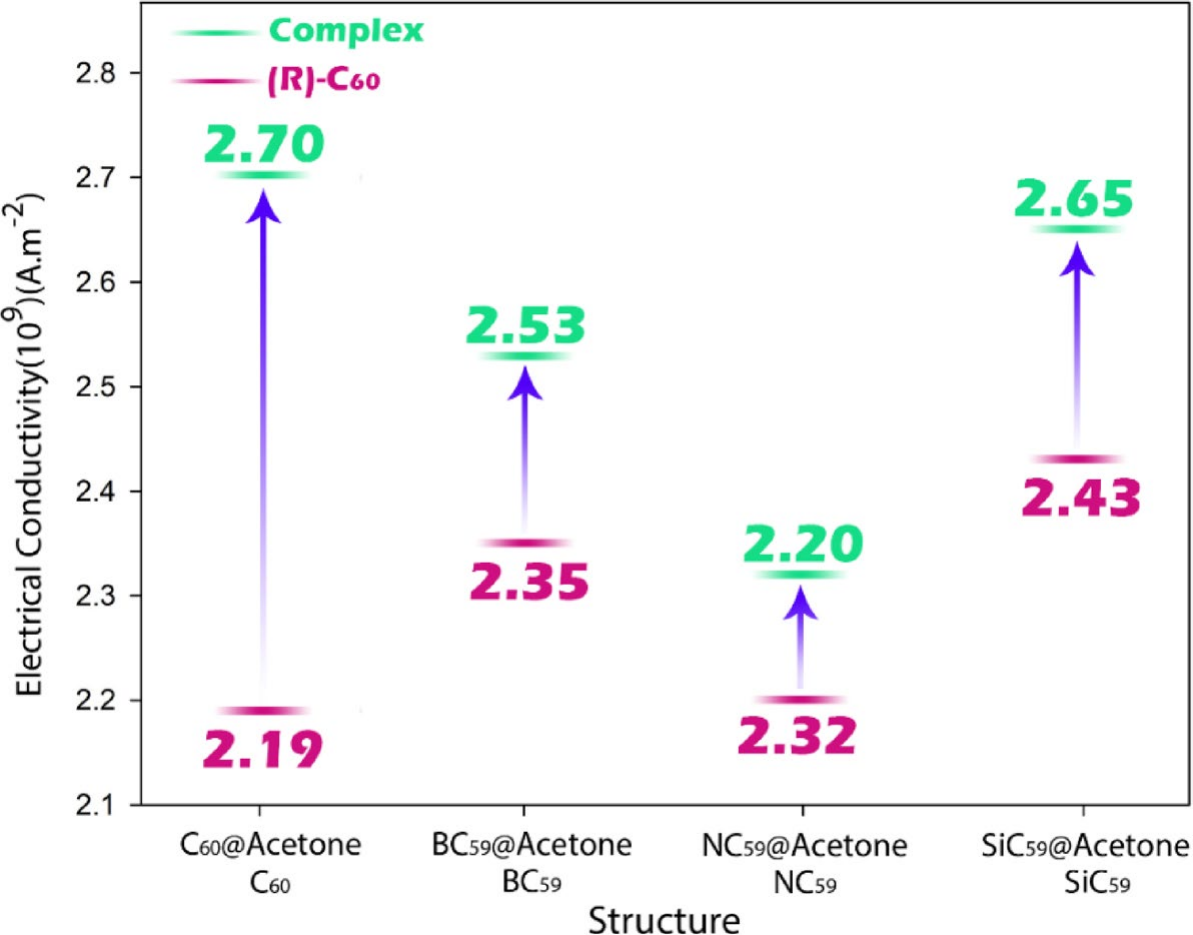
The trend of changes in electrical conductivity for each of the designed sensors in the presence and absence of acetone.

Upon acetone adsorption, all systems exhibit an increase in electrical conductivity, reflecting enhanced charge transfer and electronic coupling between the sensor surface and the analyte. This increase is most notable for C_60_ (from 2.19 × 10^9^ to 2.70 × 10^9^ A m^−2^) and SiC_59_ (from 2.43 × 10^9^ to 2.65 × 10^9^ A m^−2^), indicating a strong modulation of electronic properties in response to acetone binding. These conductivity shifts suggest a clear and measurable signal upon exposure to acetone, making them promising for electrochemical sensing applications (Fig. 9).

The adsorption energy (Eads) values further illuminate the strength of interaction between acetone and the sensor. SiC_59_@Acetone shows the highest adsorption energy (− 137.17 kJ mol^−1^), followed by BC₅₉@acetone (− 109.28 kJ mol^−1^), both indicative of strong binding and efficient recognition of the analyte. C_60_@Acetone and NC_59_@Acetone exhibit lower adsorption energies (− 35.82 and − 39.67 kJ mol^−1^, respectively), suggesting weaker interactions. While strong adsorption enhances sensitivity, overly strong binding can hinder analyte release. Therefore, it is important to balance Eads with recovery time.

Recovery time (τ), which measures how quickly the sensor returns to its original state, is in the order of 10^−10^ to 10^−11^ s for all complexes, indicating rapid desorption and excellent reversibility-an essential feature for real-time and repeated monitoring. Although SiC_59_@Acetone and BC_59_@Acetone exhibit higher adsorption energies, their recovery times (1.50 × 10^−10^ s and 1.19 × 10^−10^ s, respectively) remain suitably short, suggesting that despite strong binding, the systems are not kinetically hindered and can rapidly regenerate.

For context, J. Li et al. reported a recovery time of 5.17 s for a mesoporous 2.5% SiO2/WO3 acetone sensor at 50 ppm concentration, with a detection limit of 0.25 ppm. This value is already considered fast for semiconductor metal oxide sensors, highlighting the remarkable kinetic advantage predicted for the SiC59, and BC59 systems in this study. If experimentally realized, these materials could enable rapid regeneration and high-frequency sampling for real-time breath or environmental acetone monitoring.

From a diagnostic standpoint, these findings suggest that SiC_59_ and BC_59_ are particularly effective candidates for acetone sensing in heart failure monitoring, due to their high conductivity, strong yet reversible acetone adsorption, and short recovery times. SiC_59_@Acetone, in particular, combines the highest conductivity change, strongest binding, and fast regeneration, making it highly sensitive and practical for real-time detection. Meanwhile, C_59_@Acetone offers a more moderate interaction, suitable for reversible sensing with slightly lower sensitivity. Overall, the integration of Eads, τ, and σ data strongly supports the use of these systems (especially SiC_59_) for the electrochemical detection of acetone as a non-invasive diagnostic tool for heart failure.

#### NBO analysis

NBO analysis is crucial for sensor design as it identifies donor–acceptor interactions and quantifies their strength through the second-order perturbation energy matrix (E^2^), calculated using Eq. 12. Higher E^2^ values indicate stronger charge transfer and electron delocalization, which enhance conductivity changes and electrical signal generation upon analyte adsorption. The key electron transitions considered are σ → σ*, π → π*, LP → σ*, and LP → π*, with π → π* transitions being the most significant due to their strong influence on π-electron delocalization and frontier orbital modulation

12 $$E^{2} = \Delta E_{i,j} - q\frac{{F^{2} \left( {i,j} \right)}}{{E_{j} - E_{i} }}$$

In C_60_@Acetone, the most notable interaction occurs between the π-electrons of C1-C6 and the π*-orbitals of C7-C19, with an E^2^ value of 12.73  kJ mol^−1^, indicating strong electron delocalization. This is complemented by a σ → σ transition (2.61  kJ mol^−1^)* and a weaker lone pair (LP) donation from acetone’s oxygen (O61) into the π*-system of C60-C62 (1.79  kJ mol^−1^). These interactions collectively enhance the electronic coupling between acetone and C_60_, facilitating charge transfer and reducing the HOMO–LUMO gap (0.65 eV), which is crucial for electrochemical signal generation (Table 4).

**Table 4:** Calculated values of NBOs analysis for the studied complexes.

Complex	Donor (i)	Type	Acceptor (j)	Type	E^(2)^ kJ mol^−1^	E(j)-E(i) a.u	F(i,j) a.u
C60@Aceton	C1-C2	$\sigma$	C_1_-C_6_	${\sigma }^{*}$	2.61	1.09	0.048
	C1-C6	$\pi$	C_7_-C_19_	${\pi }^{*}$	12.73	0.25	0.051
	O61	LP (1)	C_60_-C_62_	${\pi }^{*}$	1.79	0.94	0.037
BC59@ Acetone	C1-C2	$\sigma$	C2-C3	${\sigma }^{*}$	1.28	1.08	0.047
	C1-C6	$\pi$	C2-C3	${\pi }^{*}$	6.26	0.25	0.050
	C35	LP (1)	C34-C47	${\pi }^{*}$	29.40	0.13	0.088
NC59@ Acetone	C1-C2	$\sigma$	C2-C3	${\sigma }^{*}$	1.12	1.08	0.044
	C8-C20	$\pi$	C55-C56	${\pi }^{*}$	6.29	0.26	0.051
	C47	LP (1)	C34-C35	${\pi }^{*}$	39.04	0.11	0.093
SiC59@ Acetone	C1-C2	$\sigma$	C6-C17	${\sigma }^{*}$	3.04	1.02	0.050
	C2-C3	$\pi$	C4-C5	${\pi }^{*}$	12.59	0.26	0.051
	O61	LP (1)	C36-C37	${\pi }^{*}$	0.07	0.52	0.006

The Si_C59_@Acetone system exhibits even more pronounced interactions, with a π → π transition (12.59  kJ mol^−1^)* comparable to C60 but accompanied by a stronger *σ → σ interaction (3.04  kJ mol^−1^)**. While the LP(O61) → π* contribution is minimal (0.07  kJ mol^−1^), the overall electronic restructuring in SiC59 is more dramatic due to silicon’s electrophilic nature, as evidenced by the large adsorption energy (− 137.17  kJ mol^−1^) and significant dipole moment increase (19.55 D). The shorter Si–O bond length (1.8 Å) further confirms a stronger interfacial interaction compared to C60’s C–O distance (3.2 Å), aligning with SiC_59_’s superior sensitivity.

In contrast, BC_59_@Acetone and NC_59_@Acetone show less favorable characteristics. BC_59_’s strongest interaction involves LP donation from C35 to π* orbitals (29.40  kJ mol^−1^), but this is offset by weaker π → π* transitions (6.26  kJ mol^−1^). NC59, despite having the highest LP → π* energy (39.04  kJ mol^−1^), suffers from a longer N–O distance (3.2 Å) and an increased HLG (1.66 eV), which hinder electronic responsiveness.

### NCI analysis

**Fig. 10:**
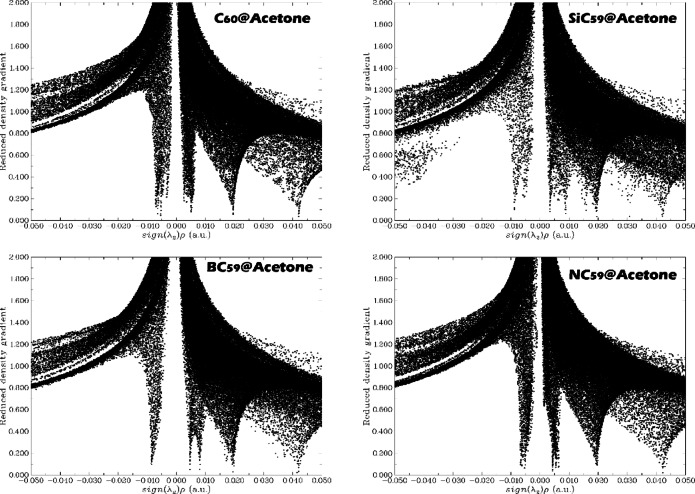
NCI analysis for the complexes designed in this work.

Non-Covalent Interaction (NCI) analysis is breakthrough technology for complex design, as it provides detailed information about weak interactions (e.g., van der Waals, hydrogen bonding, and π-π stacking) that influence the stability, selectivity, and function of sensor–analyte systems. The NCI analysis will use the reduced density gradient (RDG), electron density (ρ), and sign of the second eigenvalue of the electron density Hessian [sign(λ_2_)ρ] to quantitively and visually show attractive, repulsive, and dispersive interactions within a complex system. Thus, in each of the designed complexes, NCI graphs were computationally explored (see Fig. 10).

The RDG vs. sign(λ_2_)ρ NCI scatter plots show how each sensor-acetone pair partitions its electron density between attractive (sign(λ_2_)ρ < 0), weakly dispersive (near 0), and repulsive (sign(λ_2_)ρ > 0) regions, thereby providing a direct, geometry‑independent picture of the non‑covalent landscape (Fig. 10)^51,52^. For SiC_59_@Acetone and BC_59_@Acetone the plots display the most intense and extended spikes toward negative sign(λ_2_)ρ values, together with dense point clouds at low RDG, evidencing strong, localized attractive interactions in addition to broad dispersive contacts. This is fully consistent with their largest adsorption energies (Eads = − 137.2 and − 109.3 kJ mol^−1^, respectively) and with the short Si–O (1.8 Å) and B–O (1.6 Å) separations reported earlier. In contrast, C_60_@Acetone and especially NC_59_@Acetone exhibit distributions that are dominated by regions close to sign(λ_2_)ρ ≈ 0 with comparatively shallower excursions into the negative domain, indicating interactions that are primarily weakly attractive/dispersive; this again matches their much smaller |Eads| (− 35.8 and − 39.7 kJ mol^−1^) and long C–O/N–O distances (~ 3.2 Å) (See Fig. 10). The paucity of strong attractive signatures in NC_59_@Acetone also aligns with its electronic “hardening” (HLG increase, reduced softness) and the smallest dipole‑moment amplification among the complexes. Altogether, the NCI contours corroborate the energetic and electronic metrics: Si- and B‑doped cages form the strongest, most localized attractive contacts with acetone, while pristine C_60_ and N‑doped C_59_ interact mainly through weaker, more delocalized dispersion, mirroring the hierarchy derived from adsorption energies, bond lengths, conductivity shifts, and reactivity descriptors.

### QTAIM

Quantum Theory of Atoms in Molecules (QTAIM) is a powerful method for analyzing the nature of chemical bonds by examining the topological properties of the electron density, ρ(r). The theory identifies critical points, particularly bond critical points (BCPs), where the gradient of electron density is zero, and provides key descriptors such as the Laplacian of electron density (∇^2^ρ), kinetic energy density (G(r)), potential energy density (V(r)), and total electron energy density (Hb = V(r) + G(r)). These parameters allow the classification of bonds into covalent, ionic, hydrogen bonding, or van der Waals interactions based on the electronic structure.

Rozas et al. refined this classification for hydrogen bonds using BCP data by correlating ∇^2^ρ(r) and Hb. According to their classification: strong hydrogen bonds (Hb < 0, ∇^2^ρ(r) < 0) exhibit quasi-covalent character and high stability due to significant electron sharing; moderate hydrogen bonds (Hb > 0, ∇^2^ρ(r) < 0) are predominantly electrostatic with intermediate strength; and weak hydrogen bonds (Hb > 0, ∇^2^ρ(r) > 0) display dissociative interactions with minimal electron density overlap^53,54^. This classification framework provides a systematic way to understand bond strength and character, which is critical for predicting the stability and performance of molecular complexes in applications like electrochemical sensing and supramolecular chemistry.

**Fig. 11:**
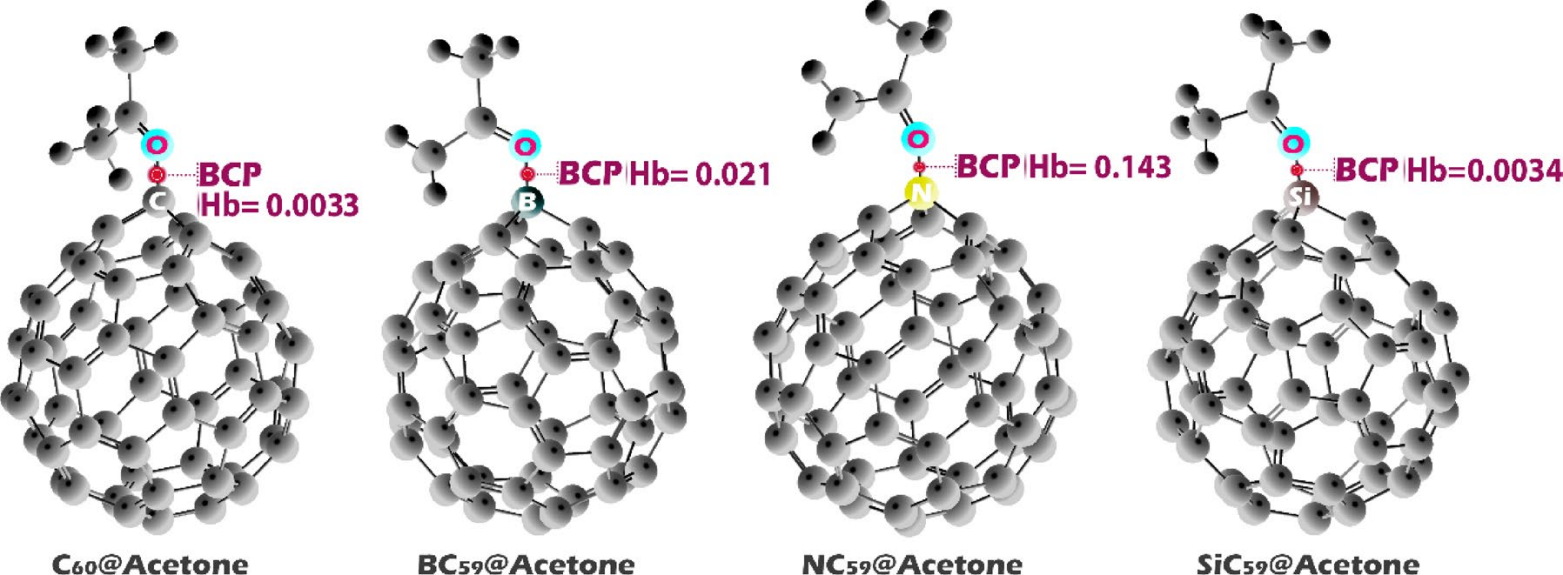
Hb values at the BCP for each of the structures studied in this work.

According to QTAIM analysis, all complexes show ∇^2^ρ(r) < 0 but Hb > 0, which places these interactions in the category of moderate hydrogen-bond-like or electrostatic interactions, as defined by Rozas et al. The electron density at the bond critical point, ρ(r), clearly differentiates the interaction strength among the complexes. BC_59_@Acetone exhibits the highest ρ(r) value (0.099 a.u.), followed by SiC_59_@Acetone (0.083 a.u.), indicating stronger and more localized interactions with acetone. In contrast, C_60_@Acetone (0.0071 a.u.) and NC_59_@Acetone (0.006 a.u.) display very low electron densities at the BCP, reflecting much weaker interactions. Similarly, the larger |V(r)| and G(r) values for BC_59_@acetone and SiC59@acetone confirm a stronger donor–acceptor interaction compared to the nearly negligible values observed in C_60_@Acetone and NC59@acetone (Fig. 11 and Table 5).

**Table 5:** QTAIM parameters including electron density (ρ(r)), kinetic energy density (G(r)), potential energy density (V(r)), Laplacian of electron density (∇^2^ρ(r)), and total energy density (Hb) at the bond critical points (BCPs) for the studied sensor–acetone complexes.

Structure	ρ(r)	G(r)	V(r)	∇^2^ρ(r)	Hb
C_60_@Acetone	0.0071	0.0047	− 0.0014	− 0.0062	0.0033
BC_59_@Acetone	0.099	0.158	0.054	− 0.103	0.212
NC_59_@Acetone	0.006	0.0048	− 0.0014	− 0.006	0.0034
SiC59@Acetone	0.083	0.125	0.018	− 0.107	0.143

These QTAIM results are consistent with the NCI analysis, which also indicated stronger non-covalent interactions for BC_59_ and SiC_59_ due to the localized electronic effects of B and Si doping, while C_60_ and NC_59_ form only weak, diffuse interactions with acetone. The combination of high ρ(r) and larger negative ∇^2^ρ(r) in the doped systems reflects a greater capacity for charge redistribution, aligning with the enhanced adsorption energies and electronic responses observed for BC_59_ and SiC_59_ in previous analyses.

### Comparison with other literature

The present study demonstrates significant advancements in acetone sensing compared to prior works, leveraging the unique properties of doped C_60_ fullerenes (B, N, Si) to achieve superior sensitivity, selectivity, and practical applicability.

Comparatively, Yong et al. investigated B_40_ and M@B_40_ (M = Li, Ba) fullerenes for acetone sensing, reporting adsorption energies of − 19.3 kJ mol^−1^ for B40 and − 25.5 kJ mol^−1^ for Li@B_40_. These values are significantly lower than those achieved in the present work, highlighting the enhanced performance of SiC_59_ (− 137.17 kJ mol^−1^) and BC_59_ (− 109.28 kJ mol^−1^). Similarly, Aasi et al. studied Pd-decorated phosphorene for acetone detection, with adsorption energies ranging from − 30.2 to − 45.8 kJ mol^−1^, which are still weaker than the interactions observed for SiC_59_ and BC_59_. Würger et al. explored acetone adsorption on rutile TiO_2_, reporting an Eads of − 52.0 kJ mol^−1^, which, while stronger than some literature examples, remains inferior to the results obtained here.

This work advances acetone sensing by combining the versatility of doped fullerenes with robust computational validation, offering a transformative approach for heart failure monitoring and environmental remediation.

### Future outlook

The development of fullerene C_60_-based sensors for acetone detection presents a promising avenue for non-invasive heart failure diagnosis and environmental monitoring. Future research should focus on experimental validation of the computational findings to confirm the sensitivity, selectivity, and practical applicability of these sensors. Additionally, exploring the scalability and cost-effectiveness of synthesizing doped C_60_ materials will be crucial for real-world implementation. Further studies could explore the integration of these sensors into wearable or portable devices, allowing monitoring of acetone levels in exhaled breath for early detection of heart failure. Advances in nanotechnology and material science may also open doors to multifunctional platforms that combine sensing with therapeutic capabilities, such as controlled drug release. Collaborative efforts between computational chemists, material scientists, and medical researchers will be essential to translate these theoretical insights into transformative diagnostic tools and environmental solutions.

## Conclusion

This study successfully demonstrated the dual functionality of C_60_ fullerenes doped with silicon (Si), nitrogen (N), and boron (B) for medical diagnostic and environmental remediation applications using DFT and QTAIM theories. The electronic structure analysis showed significant modulation of energy gaps upon acetone adsorption. Pristine C_60_ displayed an energy gap reduction from 1.68 eV to 0.65 eV (ΔHLG = − 1.03 eV), while SiC_59_ showed a substantial decrease from 1.18 eV to 0.74 eV (ΔHLG = − 0.44 eV). These pronounced reductions in HOMO–LUMO gaps indicate enhanced electronic sensitivity crucial for sensor applications. The dipole moment analysis revealed even more striking changes, with SiC59 exhibiting a remarkable increase from 0.35 D to 19.55 D (Δμ =  + 19.20 D) upon acetone binding, demonstrating significant charge redistribution that facilitates measurable signal generation in sensing devices. For practical applications, the adsorption characteristics proved particularly noteworthy. SiC_59_ showed the strongest acetone adsorption energy (− 137.17 kJ mol^−1^) among all tested systems, while maintaining an excellent recovery time of 1.50 × 10^−10^ s. This combination of strong binding and rapid desorption makes it ideal for both sensitive detection and reusable applications. The electrical conductivity measurements further supported these findings, with SiC_59_ showing a conductivity increase from 2.43 × 10^9^ A m^−2^ to 2.65 × 10^9^ A m^−2^ upon acetone exposure, indicating its potential for real-time monitoring applications. The comparative analysis clearly established the superiority of Si-doped systems over both pristine C_60_ and other doped variants. While boron-doped C59 (BC_59_) showed promising results with an adsorption energy of − 109.28 kJ.mol^−1^ and conductivity increase to 2.53 × 10^9^ A m^−2^, nitrogen-doped C59 (NC_59_) proved less effective, actually showing an increased energy gap and decreased conductivity upon acetone interaction. The high sensitivity, strong binding, and rapid recovery in SiC_59_ present an exciting opportunity for developing advanced multifunctional materials that can address both healthcare and environmental challenges simultaneously. To translate the computational findings into practical applications, future work should focus on the experimental fabrication of doped fullerene C60-based sensors. Potential fabrication strategies include chemical vapor deposition (CVD) for controlled heteroatom doping, solution-phase self-assembly for scalable synthesis of nanostructured fullerene frameworks, and thin-film deposition methods (such as spin coating or drop casting) for sensor device integration. These techniques are well-established in nanomaterial processing and can enable the realization of high-performance, reusable, and portable acetone sensors suitable for both clinical diagnostics and environmental monitoring.

## Data Availability

The datasets used and/or analysed during the current study available from the corresponding author on reasonable request.
